# Neurovascular unit dysfunction with blood-brain barrier hyperpermeability contributes to major depressive disorder: a review of clinical and experimental evidence

**DOI:** 10.1186/1742-2094-10-142

**Published:** 2013-12-01

**Authors:** Souhel Najjar, Daniel M Pearlman, Orrin Devinsky, Amanda Najjar, David Zagzag

**Affiliations:** 1 Department of Neurology, Neuroinflammation Research Group, Epilepsy Center Division, NYU School of Medicine, New York, NY 10016, USA; 2 Department of Neurology, NYU Comprehensive Epilepsy Center, NYU School of Medicine, New York, NY 10016, USA; 3 The Dartmouth Institute for Health Policy and Clinical Practice, Geisel School of Medicine at Dartmouth, Lebanon, NH 03766, USA; 4 Department of Pathology, Division of Neuropathology, NYU School of Medicine, New York, NY 10016, USA; 5 Department of Neurosurgery, NYU School of Medicine, New York, NY 10016, USA

**Keywords:** Major depressive disorder, Blood-brain barrier, Neurovascular unit, Neuroinflammation, Oxidative stress, Nitric oxide synthase, eNOS uncoupling, Peroxynitrite

## Abstract

About one-third of people with major depressive disorder (MDD) fail at least two antidepressant drug trials at 1 year. Together with clinical and experimental evidence indicating that the pathophysiology of MDD is multifactorial, this observation underscores the importance of elucidating mechanisms beyond monoaminergic dysregulation that can contribute to the genesis and persistence of MDD. Oxidative stress and neuroinflammation are mechanistically linked to the presence of neurovascular dysfunction with blood-brain barrier (BBB) hyperpermeability in selected neurological disorders, such as stroke, epilepsy, multiple sclerosis, traumatic brain injury, and Alzheimer’s disease. In contrast to other major psychiatric disorders, MDD is frequently comorbid with such neurological disorders and constitutes an independent risk factor for morbidity and mortality in disorders characterized by vascular endothelial dysfunction (cardiovascular disease and diabetes mellitus). Oxidative stress and neuroinflammation are implicated in the neurobiology of MDD. More recent evidence links neurovascular dysfunction with BBB hyperpermeability to MDD without neurological comorbidity. We review this emerging literature and present a theoretical integration between these abnormalities to those involving oxidative stress and neuroinflammation in MDD. We discuss our hypothesis that alterations in endothelial nitric oxide levels and endothelial nitric oxide synthase uncoupling are central mechanistic links in this regard. Understanding the contribution of neurovascular dysfunction with BBB hyperpermeability to the pathophysiology of MDD may help to identify novel therapeutic and preventative approaches.

## Background

Major depressive disorder (MDD) is the second leading global cause of years lived with disability [1], with about one-third of patients with MDD failing two or more conventional antidepressant drug trials within the first year of treatment [2,3]. Current evidence suggests that the pathophysiology of MDD is multifactorial, involving heterogeneous and inter-related mechanisms that affect genetic, neurotransmitter, immune, oxidative, and inflammatory systems [4]. Supporting this interpretation, whereas biomarkers for individual abnormalities possess limited predictive validity for MDD, the predictive validity of several composite biomarker assays is particularly high [5]. For example, one study of 36 patients with MDD showed that a compositive biomarker test—comprising nine individual biomarker assays (α1 antitrypsin, apolipoprotein CIII, myeloperoxidase, soluble tumor necrosis factor α (TNFα) receptor type II, epidermal growth factor, cortisol, brain-derived neurotropic factor, prolactin, and resistin)—had 91.7% sensitivity and 81.3% specificity for MDD [6]. A follow-up study involving a distinct sample of 34 MDD patients and using the same composite assay, replicated these results with a high degree of precision: 91.1% sensitivity, 81.0% specificity [6].

**Figure 1:**
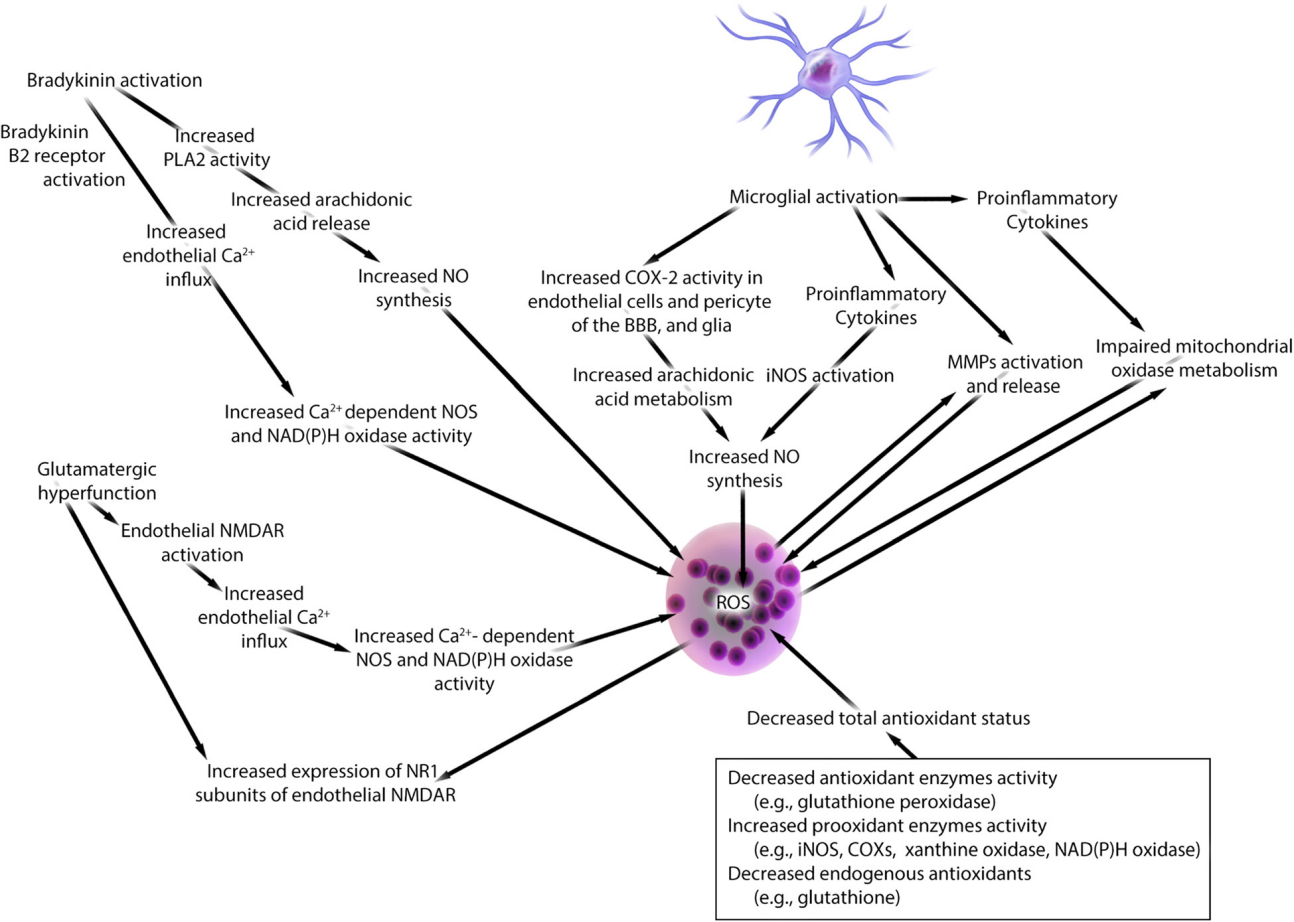
**Putative mechanisms involving the synthesis of reactive oxygen species (ROS) and their bidirectional interaction with neuroinflammation in major depressive disorder.** This figure shows potential mechanistic links among ROS, inflammation, and hyperglutamatergia. Abbreviations: BBB, blood-brain barrier; COX2, cyclo-oxygenase 2; CRH, corticotropin-releasing hormone; eNOS, endothelial nitric oxide synthase; iNOS, inducible nitric oxide synthase; MMP, matrix metalloproteinase; NAD(P)H, nicotinamide adenosine dinucleotide phosphate; NMDAR, *N*-methyl-D-aspartate receptor; NO, nitric oxide; PLA2, phospholipase A2.

Oxidative stress and neuroinflammation are implicated in the neurobiology of MDD [7-14] (recently reviewed by our group [4,15-19]). Neuropathological studies comparing brain tissue from individuals with MDD to that from non-depressed controls have documented associations between MDD and (a) decreased levels of antioxidants, such as glutathione [11,15,16] and (b) increased levels of lipid peroxidation end products, such as 4-hydroxy-2-nonenal [8]. Studies assessing peripheral markers of oxidative stress have reported similar findings, including: (a) altered activity of antioxidant enzymes, such as glutathione peroxidase, catalase, superoxide dismutase 1, (b) increased activity of pro-oxidant enzymes such as, xanthine oxidase, (c) increased activity of inducible nitric oxide synthase (iNOS) in leukocytes, (d) increased levels of superoxide (O_2_^-^), and (e) increased levels of 8-hydroxy-2-deoxyguanosine (a marker for oxidative damage to DNA) [11,12]. Evidence deriving from genetic, neuropathological, cerebrospinal fluid, and serum studies in humans with MDD and from animal models of depressive-like behavior and chronic stress reveal numerous neuroinflammatory abnormalities in MDD, including [4]: (a) microglial activation [17-19], (b) astroglial loss and activation [20,21], (c) upregulated ratios of T helper 1 (Th1) cells and proinflammatory cytokines [22-24], and (d) decreased CD4^+^CD25^+^FOXP3^+^ regulatory T (T_Reg_) cell counts [25]. Both oxidative stress and neuroinflammation may contribute to decreased serotonergic and increased glutamatergic tone, and increased glutamatergic tone may in turn contribute to oxidative stress and neuroinflammation in a positive feedback loop [4]. In addition, experimental evidence suggests that increased reactive oxygen species (ROS) synthesis (oxidative stress) and neuroinflammation themselves exhibit a bidirectional relationship (Figure 1). Indeed, ROS can activate microglia and increase proinflammatory cytokine synthesis—for example, by stimulating transcription factor nuclear factor κB (NFκB)—whereas activated microglia and proinflammatory cytokines can in turn perpetuate oxidative stress [8,11,26-28].

Collectively, data from postmortem neuropathological human studies and *in vivo* neuroimaging human and animal studies provide strong evidence of neurovascular unit dysfunction with blood-brain barrier (BBB) hyperpermeability in association with oxidative stress and neuroinflammation in selected neurological disorders, such as stroke, epilepsy, Alzheimer’s disease, traumatic brain injury, and multiple sclerosis [29-43] (Table 1). In these disorders, BBB breakdown, oxidative stress, and inflammation are thought to impair neuronal function [44]. MDD, in contrast to other major psychiatric disorders, is frequently comorbid with such neurological disorders as well as disorders characterized by vascular endothelial dysfunction, such as cardiovascular disease and diabetes mellitus [45-52]. Whether neurovascular dysfunction with BBB hyperpermeability occurs in primary MDD (without neurological comorbidity), however, remains less clear.

**Table 1:** Putative mechanisms of neurovascular dysfunction and blood–brain barrier hyperpermeability in major depressive disorder in the context of established mechanisms in various neurological disorders

**Mechanisms**	**Major depressive disorder**				**Neurological disorders**	
	**Human**	**Sources**	**Animal**	**Sources**	**Human**	**Sources**
**Oxidative stress**						
eNOS uncoupling, decreased NO	■	[53-59]	■	[60]	●	[61-63]
Increased ROS synthesis	●	[10,14-16,64-84]	●	[64,85]	●	[42,63,86]
Cerebral hypoperfusion	●	[87-91]	N/A	…	●	[92-96]
MMP activation	■	[97]	?	…	●	[39,42,98-101]
Decreased E-cadherin activity	?	…	?	…	?^a^	[38,102]
Tight junction alteration	?	…	?	…	●	[31,38,41,103-106]
Endothelial cytoskeletal alteration	?	…	?	…	●	[31]
Increased NMDAR expression^b^		[107-111]	●	[40]	●	[112]
Mitochondrial alterations	●	[65,113-121]	●	[65,122]	●	[11,123-125]
**Neuroinflammation**						
Astroglial loss	●	[20,21,126-133]	●	[134-138]	●	[139-141]
Decreased AQP4	●	[142]	●	[143]	●	[144-146]
Microglial activation	●	[18,147,148]	●	[149-152]	●	[42,98,153,154]
Proinflammatory cytokines	●	[23,155]	●	[156,157]	●	[42,98]
Bradykinin alteration	●^c^	[158]	●	[159]	●	[159,160]
Hyperglutamatergia	●	[4,161-163]	●	[164]	●	[165-167]
Mast cell activation	●^c^	[168,169]	?	…	●	[170,171]
Increased ICAM-1 and VCAM-1		[172-174]	?	…	●	[175-178]
**Other mechanisms**						
Increased P-glycoprotein activity	●	[179,180]	●	[181]	●	[179]

Symbol key: ●, documented in the central nervous system in major depressive disorder; ■, not documented in the central nervous system, but associated with major depressive disorder; ?, insufficient data; , mixed evidence.

Abbreviations: *AQP4,* aquaporin 4; *eNOS*, endothelial nitric oxide synthase; *ICAM-1*, intercellular adhesion molecule 1; *NMDAR, N*-methyl-D-aspartate receptor; *MMP,* matrix metalloproteinases; *ROS*, reactive oxygen species; *VCAM-1*, vascular cell adhesion molecule 1.

a. Refers to data that has only been shown in animal models.

b. Refers to human data in major depressive disorder refers to increased NMDAR expression that was not specific to the endothelium. Human data of NMDAR subunit composition alteration in neurological disorders was shown in cultured human blood–brain barrier endothelial cells. Animal data refer to increased cerebrovascular endothelial NMDAR subunit 1 (NR1) expression upon exposure to oxidative stress (this was not a depressive-like behavior or chronic stress animal model, though this evidence may be relevant to MDD where oxidative stress is documented).

c. Refers to abnormalities for which only limited data exists.

Shalev and colleagues have previously reviewed evidence through 2009 linking BBB hyperpermeability to psychiatric disorders generally [168]. We review emerging clinical and experimental evidence implicating oxidative stress, eNOS uncoupling, and reduced endothelial NO levels in the pathophysiology of peripheral vascular endothelial dysfunction associated with MDD. We present a theoretical integration of human and animal data linking these mechanisms and those involving neuroinflammation to findings suggesting that neurovascular dysfunction can occur in primary MDD. We also discuss putative links between neurovascular dysfunction with BBB hyperpermeability and neuronal signaling abnormalities in MDD.

### Neurovascular unit dysfunction

The neurovascular unit consists of cerebral microvessels, glial cells (astroglia, microglia, oligodendroglia), and neurons. It is the epicenter of several tightly controlled, dynamic, and complex cellular interactions between glia and neurons, and the coupling of neuronal activity with endothelium-dependent cerebral blood flow [33]. Evidence of an association between MDD and neurovascular dysfunction is indirect, deriving primarily from studies assessing peripheral vascular endothelial dysfunction in MDD and from epidemiological data associating MDD with vascular disorders.

One method for evaluating endothelial dysfunction involves measuring the relative uptake ratio (RUR) of blood flow in the brachial artery after hyperemic challenge via dynamic nuclear imaging. RUR is a measure of the vascular dilatory response whereby a lower RUR implies poorer vascular endothelial function. In a prospective cohort involving 23 patients with MDD, 23 with minor depressive disorder, and 277 non-depressed controls, the mean RUR was significantly lower in participants with MDD (unadjusted mean = 3.13, SD = 1.51) or minor depressive disorder (unadjusted mean = 3.38, SD = 1.00) compared with non-depressed controls (unadjusted mean = 4.22, SD = 1.74) (*F* = 6.68, *P* = 0.001) [182]. This effect remained statistically significant after adjusting for age, sex, socioeconomic factors, medical comorbidity, and medications (*F* = 5.19, *P* = 0.006) [182]. One study evaluating endothelial proapoptotic activity, defined as the percentage of apoptotic nuclei in human umbilical vein endothelial cells, found a significantly increased percentage of proapoptotic nuclei in participants with MDD compared with non-depressed controls (4.4% vs 2.3%, *P* ≤ 0.001) [183]. This finding remained statistically significant after adjusting for age and cardiovascular comorbidity.

Linking vascular endothelial dysfunction to MDD, epidemiological studies reveal a strong and bidirectional association between MDD and medical conditions characterized by vascular endothelial pathology [184]. A recent meta-analysis involving 16,221 study participants found a significantly increased risk of MDD among individuals with major vascular diseases compared with those without vascular disease: diabetes (odds ratio (OR) 1.51, 95% confidence interval (CI) 1.30 to 1.76, *P* < 0.0005, 15 studies), cardiovascular disease (OR 1.76, 95% CI 1.08 to 1.80, *P* < 0.0005, 10 studies), and stroke (OR 2.11, 95% CI 1.61 to 2.77, *P* < 0.0005, 10 studies) [45]. The same meta-analysis also found that MDD was more common among individuals with two or more classic risk factors for vascular disease compared with those with one or no risk factors (OR 1.49, 95% CI 1.27 to 1.7, *P* < 0.0005, 18 studies) [45]. These findings remained robust after statistical adjustments for chronic illness and disability. Results from meta-analyses having assessed the association from the reverse direction, indicate that MDD is not only an independent risk factor for cardiovascular disease (relative risk (RR) 2.69, 95% CI 1.63 to 4.43, *P* < 0*.*001, 11 studies) [49], but is also associated with a 3-fold increased cardiovascular disease mortality rate (OR 2.61, 95% CI 1.53 to 4.47, *P* = 0.0004) [48]. Related studies report similar findings [50-52].

### Blood–brain barrier unit hyperpermeability

The BBB consists of the neurovascular endothelium, extracellular matrix basal lamina, and astrocytic end-feet processes. The BBB secures the brain’s immune-privileged status by restricting the entry of peripheral inflammatory mediators (for example, cytokines, antibodies), which can impair neurotransmission [37,168,185,186]. Neurovascular endothelial cells regulate influx of essential nutrients, efflux of toxic substances, ionic homeostasis of brain interstitial fluid, and prevent brain influx of peripheral neuroactive substances, neurotransmitters, and water-soluble molecules [185]. Evidence of an association between BBB hyperpermeability and MDD derives mainly from studies having assessed cerebrospinal fluid (CSF)-to-serum ratios of various molecules, as well as evaluations concerning P-glycoprotein.

Evidence of an elevated CSF-to-serum albumin ratio in some MDD patients is suggestive of mild hyperpermeability of blood-brain and/or blood-CSF barriers [186,187]. A cross-sectional study of elderly women without dementia (11 MDD, 3 dysthymia, 70 non-depressed controls) found an elevated mean CSF-to-serum albumin ratio among those with MDD or dysthymia relative to non-depressed controls (7.1 × 10^-3^ vs 5.4 × 10^-3^, age-adjusted *P* < 0.015) [186]. Another study (24 affective disorders, 4,100 age-matched controls) found an increased mean CSF-to-serum albumin ratio among 37.5% of the affective disorder group (9 of 24); this value was 22% to 89% above the upper limit of healthy age-matched controls (8.7 × 10^-3^ vs 5.0 × 10^-3^) [187]. A third study (99 MDD) found that increased CSF-to-serum ratios of albumin and urate were positively associated with EEG slowing (a measure of cerebral dysfunction) and suicidality [188]. Elevated levels of S100B protein (a marker of glial activation) [189,190] and proinflammatory cytokines [23,191] in the serum, CSF, and neuropathological specimens from persons with MDD may be related to increased permeability of blood-brain and blood-CSF barriers. Elevated levels of these molecules may reflect their increased synthesis and increased efflux from (a) brain parenchyma into the blood (BBB hyperpermeability) [168,184], and (b) blood into the CSF (blood-CSF hyperpermeability).

Alteration of BBB endothelial expression of P-glycoprotein (a multidrug efflux transporter) is documented in some persons with MDD [192]. Reduced expression or function of P-glycoprotein may facilitate BBB permeability to neurotoxic substances [192]. Positron emission tomography (PET) utilizing the [(11)C]-verapamil radioligand for P-glycoprotein in humans with MDD and in Wistar rats exhibiting depressive-like behavior showed that chronic stress exposure and administration of antidepressants inhibited and enhanced P-glycoprotein function, respectively [179,181]. A human genetics study (631 MDD, 110 non-depressed controls) revealed a significant association between alteration of the P-glycoprotein encoding gene ATP-binding cassette, subfamily B member 1 (ABCB1) and MDD (*P* = 0.034) [180].

### Theoretical integration with oxidative and neuroinflammatory mechanisms

### Oxidative stress

Common ROS include superoxide (O_2_^-^), hydroxyl radical (HO^-^), hydrogen peroxide (H_2_O_2_^-^), and peroxynitrite (ONOO^-^). ONOO^-^ is a highly reactive oxidant generated by the reaction of nitric oxide (NO) with O_2_^-^[8,15,123]. The brain is particularly susceptible to oxidative stress due to high levels of peroxidizable polyunsaturated fatty acids and transition minerals (reduced form) that induce lipid peroxidation and convert H_2_O_2_^-^ to HO^-^; additionally, the brain’s oxygen demand is particularly high and the presence of antioxidant defense mechanisms is relatively limited [8,11,12].

**Figure 2:**
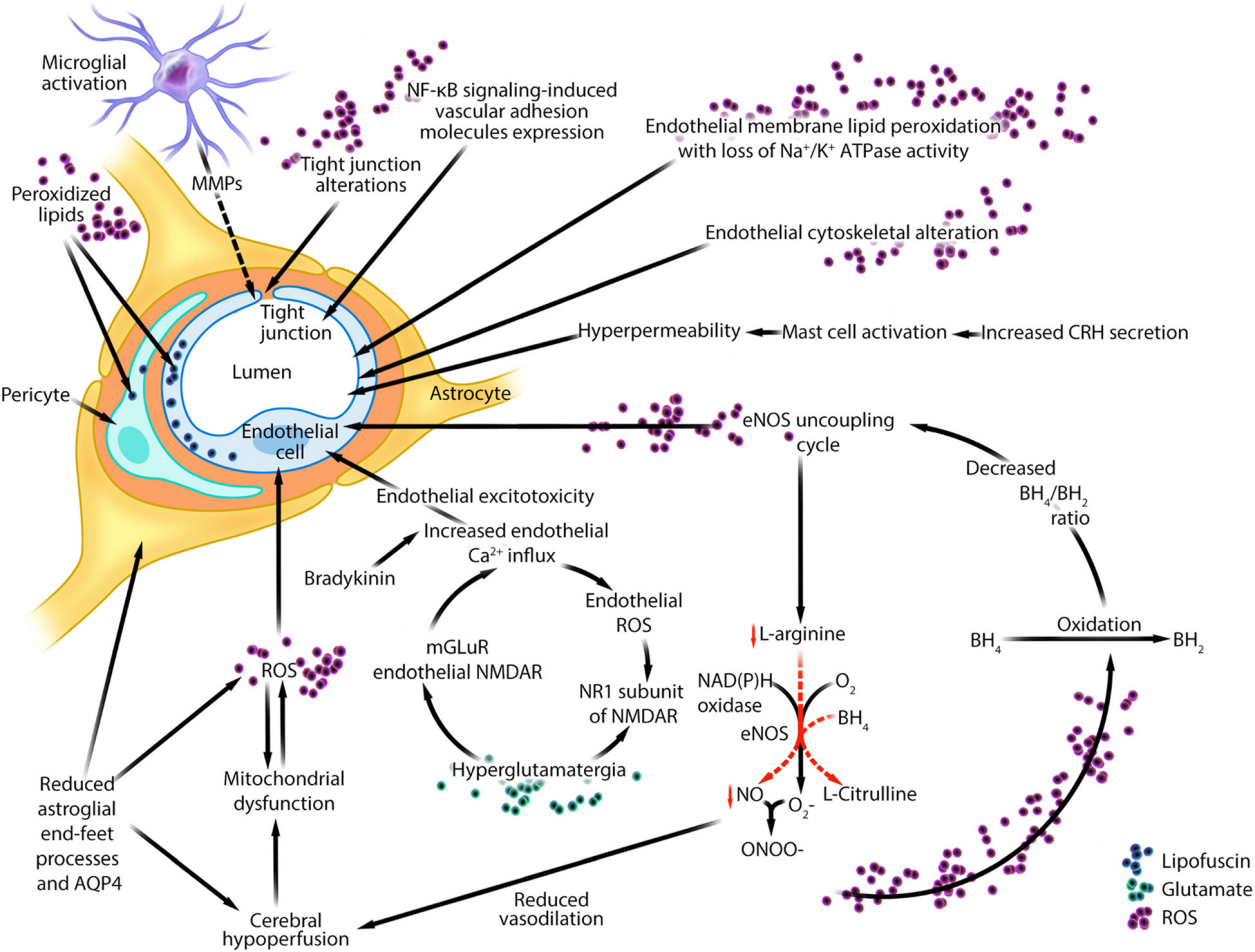
**Theoretical integration of the human and animal data linking oxidative stress, eNOS uncoupling, low endothelial NO levels, and neuroinflammation to indirect evidence of functional and structural abnormalities of neurovascular unit in major depressive disorder.** Adapted with permission from Abbott *et al*., [185]. This figure describes several putative mechanisms involving neuroinflammation, oxidative stress, endothelial nitric oxide synthase uncoupling, and hyperglutamatergia, as well as their relationships to indirect evidence of neurovascular dysfunction in MDD. Neurovascular endothelial lipofuscin granule accumulation is a marker of endothelial oxidative stress, which we recently documented by ultrastructural analysis of cerebral microvasculature in brain biopsy from a patient with chronic refractory MDD [90]. Abbreviations: AQP4, aquaporin 4; BH_2_: dihydrobiopterin; BH_4_, tetrahydrobiopterin; CRH, corticotropin-releasing hormone; eNOS, endothelial nitric oxide synthase; mGluR, metabotropic glutamate receptor; MDD, major depressive disorder; MMP, matrix metalloproteinase; NAD(P)H, nicotinamide adenosine dinucleotide phosphate; Na^+^/K^+^ ATPase, sodium-potassium adenosine triphosphatase; NFκB, nuclear factor κB; NMDAR, *N*-methyl-d-aspartate receptor; NO, nitric oxide, ONOO^-^, peroxynitrite; O_2_^-^, superoxide; ROS, reactive oxygen species.

Although ROS can limit injury and promote recovery at low levels, ROS facilitate oxidative injury at high levels by damaging biological macromolecules, such as lipids, proteins, and DNA [8,11,12]. We hypothesize that oxidative stress associated with MDD may impair neurovascular function through several mechanisms, with an emphasis on mechanisms that can shift the functional balance between beneficial endothelial nitric oxide synthase (eNOS)-generated NO versus harmful eNOS-generated O_2_^-^ (Figure 2 and Table 1).

NO has been termed ‘Janus faced’ owing to its ability to either protect vascular endothelial cell function in some instances, while impairing it in others [193]. These differential effects of NO are primarily determined by its cellular source (non-endothelial vs endothelial) and concentration (high vs low). NOS isoforms regulate NO synthesis in the brain. Of these, one is constitutively expressed in endothelial cells and astrocytes (eNOS) [194,195] (that is, eNOS), and another is expressed in neurons (neuronal NOS (nNOS)).

eNOS regulates vascular smooth muscle tone and nNOS modulates neurotransmission. The expression of a third NOS isoform, iNOS, occurs in glial and inflammatory cells and is induced by pathological inflammatory states, such as following trauma [38]. More recently, a fourth NOS isoform was described, mitochondrial (mtNOS), which is an eNOS-like isoform that is constitutively expressed in the inner mitochondrial membrane [196,197]. When combined with O_2_^-^, NO produced by non-endothelial cellular sources (as regulated by nNOS, iNOS) can impair the vascular endothelium and disrupt BBB integrity [38,53]. nNOS activity itself is positively regulated by Ca^2+^ influx [198], whereas iNOS activity is positively regulated by proinflammatory cytokine [199] and NFκB signaling [200].

NO produced by endothelial cells (as regulated by eNOS) increases cellular levels of cyclic guanosine monophosphate, which can increase cerebral blood flow via mechanisms involving endothelium-dependent vasodilation and platelet aggregation inhibition [38,53,201]. *In vitro* studies showed that endothelial-derived NO may dilate cerebral vessels by inhibiting the synthesis of 20-hydroxyeicostetranoic acid—an arachidonic acid metabolite that promotes vasoconstriction [202,203]. Endothelial-derived NO can also limit endothelial vascular oxidative stress injury by scavenging free radicals [38,53]. Endothelial eNOS mediates NO synthesis via oxidative conversion of l-arginine to l-citrulline. Activity of eNOS is modulated by several factors, including endothelial levels of Ca^2+^, arginine (eNOS substrate) [204], as well as tetrahydrobiopterin (BH_4_) (eNOS cofactor) [53-55,201,205,206] (Figure 2). Downregulation of eNOS activity can decrease endothelial NO levels, potentially resulting in (a) reduced cerebral blood flow, (b) increased platelet aggregation, which may contribute to the increased risk of cardiovascular disease in MDD, (c) increased oxidative stress, and (d) decreased vascular reactivity [38,53,201].

Under oxidative conditions, such as those associated with MDD [4,8,11,12,15] (Figure 1), endothelial levels of BH_4_ are decreased due to increased oxidative conversion of BH_4_ to dihydrobiopterin (BH_2_). Decreased endothelial levels of BH_4_ and increased endothelial levels of BH_2_ (which can also reduce BH_4_ binding to eNOS) uncouple l-arginine oxidation from the electron transfer process and shift the eNOS substrate from l-arginine to molecular oxygen (that is, eNOS uncoupling), thereby promoting the synthesis of harmful O_2_^-^ instead of beneficial NO [53-55,205,207,208]. Once formed, O_2_^-^ reacts with residual NO (still being produced at a lower rate) to form ONOO^- ^[205]. ONOO^-^ in turn oxidizes BH_4_, thereby further decreasing its levels in a positive feedback loop [54,205] (Figure 2).

Data from *in vitro* animal models of neurological disorders show that upregulation of iNOS and nNOS expression and downregulation of eNOS expression can worsen neuronal injury [209-213]. In murine models of ischemic stroke, knocking out iNOS and nNOS decreased the size of infarct while knocking out eNOS expanded infracted zone, compared to wild-type mice [214,215]. In animal models of traumatic brain injury, increased levels of endothelial ONOO^-^ are associated with BBB breakdown and neurobehavioral deficits [209]; additionally, treatment with the antioxidant *S*-nitrosoglutathione enhances neural reparative mechanisms and improves neurovascular unit function by decreasing endothelial ONOO^-^ synthesis [209].

Clinical and experimental studies suggest that eNOS uncoupling can contribute to vascular endothelial dysfunction in both cardiovascular diseases and MDD [4,53-55,182,205,206,216]. In cardiovascular diseases, eNOS uncoupling-mediated endothelial dysfunction is thought to result from (a) increased O_2_^-^ synthesis (through an NAD(P)H oxidase-dependent mechanism), (b) increased ONOO^-^ formation, and (c) decreased BH_4_ levels [54,55,182,206]. In MDD, however, the potential contribution of eNOS uncoupling to vascular endothelial dysfunction is inferred from less direct evidence. For example, several clinical studies of persons with MDD have shown significant reductions in eNOS activity and NO levels in platelets and sera, respectively [53-57]. In a study of 57 MDD patients randomized to either citalopram (*n* = 36) or placebo (*n* = 21), a 3-month trial of citalopram was associated with a statistically significant increase in serum NO levels compared to placebo (*P* = 0.005) [58]. Another study involving a 2-month trial of paroxetine reproduced similar results [59]. Fluoxetine treatment in a chronic stress mouse model restored previously deficient aortic endothelial NO levels [60], suggesting that eNOS uncoupling may not only occur in MDD, but also that eNOS recoupling may be one of the mechanisms by which antidepressants exert their therapeutic effects [8,11,14].

The antidepressant effect of l-methylfolate, which can reverse eNOS uncoupling *in vitro* via upregulating BH_4_ synthesis [206], suggests that eNOS uncoupling contributes to the neurobiology of MDD. A randomized controlled trial showed that adding l-methylfolate at 15 mg/day, but not at 7.5 mg/day, to a stable regimen of selective serotonin reuptake inhibitors (SSRIs) had superior efficacy to SSRIs plus placebo [217]. Although the authors attributed BH_4_ augmenting the antidepressant effects of SSRIs to direct activation of the rate-limiting enzymes of monoamine synthesis (serotonin, norepinephrine, dopamine), we suggest that these effects may also be related to the ability of BH_4_ to reverse eNOS uncoupling.

Although regionally selective (thalamic nuclei, prefrontal, anterior cingulate, temporal, and occipital cortices) cerebral hypoperfusion abnormalities in MDD have traditionally been attributed to depressed mood states and reduced neuronal activity [87-91][208], these findings may also be related to eNOS uncoupling [65,113,114,218] (Figure 2). Sustained cerebral hypoperfusion can impair endothelial mitochondrial oxidative function, resulting in increased synthesis of endothelial ROS [219-222]. ROS can in turn promote eNOS uncoupling, leading to reduced vasodilatory endothelial NO levels and cerebral hypoperfusion in a positive feedback loop [54,55,182,206]. In addition, SSRIs have been shown to induce vasodilation through eNOS-mediated downregulation of NO [223]. We recently reported a case of chronic and refractory MDD with moderately severe bifrontal cerebral hypoperfusion (seen via single photon emission tomography (SPECT)) associated with lipofuscin granule accumulation (a marker of oxidative stress [224-228]) (Figure 2) identified exclusively within the neurovascular unit (predominately within the endothelium) [90]; restoration of cerebral hypoperfusion in temporal association with intravenous immunoglobulin and minocycline therapy was accompanied with significant improvement of depressive symptoms, after more than 20 years of refractoriness to conventional psychiatric treatments [90]. We suggest that eNOS uncoupling may occur in MDD primarily as the result of non-heritable factors such as oxidative mechanisms. Indeed, several genetic studies show a non-significant association between eNOS gene polymorphisms and MDD [229,230].

Under oxidative conditions, BBB endothelial cells are not only the source of harmful eNOS uncoupling, but also can be the target of oxidative damage [39]. In neurological disorders associated with neurovascular dysfunction, oxidative stress can also increase BBB permeability through several mechanisms (Table 1), which include: (a) activation of metalloproteinase (MMP)-2/9 directly or indirectly through proinflammatory cytokines [39]; (b) downregulation of endothelial expression of E-cadherin [38]; (c) alteration of the expression, distribution, and phosphorylation of BBB tight junction proteins (for example, claudin, occluding, ZO proteins) by molecules such as phosphatidylinositol-3-kinase γ [38,41,103,104]; (d) alteration of endothelial cytoskeletal structure; (e) induction of endothelial NMDAR subunit expression such as NMDA receptor subunit 1 (NR1) subunit, leading endothelial excitotoxicity [40]; and (f) impairment of vascular endothelial mitochondrial oxidative metabolism [11,123]. The relevance of these mechanisms to the neurobiology of MDD, however, remains unclear (Table 1 and Figure 2).

### Neuroinflammation

Neuroinflammation may impair neurovascular function and increase BBB permeability in MDD [4,168] (Figure 2 and Table 1). Astroglial cells are an integral part of the neurovascular unit. They are involved in regulating blood flow, BBB permeability, energy metabolism, and neuronal signaling [4,184]. Astroglial loss has been consistently documented in functionally relevant areas (prefrontal and cingulate cortices, amygdala, hippocampus) among persons with MDD [4,142,168,231-236]. Other studies have documented decreased expression of the astroglial end-feet process water channel, aquaporin 4 (AQP4) in the orbitofrontal cortical gray matter (but not white matter) of individuals with MDD relative to non-depressed controls [142]. Animal models of depressive-like behavior also found decreased AQP4 density in association with oxidative stress [143]. Decreased AQP4 density may impair critical glial-vascular homeostatic pathways within the neurovascular unit and increase BBB permeability (Figure 2). Reduced AQP4 density may also contribute to cerebral perfusion and metabolic abnormalities detected by SPECT and PET imaging in human MDD [184].

Microglia provide immune surveillance and regulate developmental synaptic pruning of the brain [237]. Although transient microglial activation and proliferation (MAP) can limit neuronal injury and enhance recovery (beneficial phenotype), persistent MAP can induce and exacerbate neuronal injury (harmful phenotype) [238]. Harmful MAP is implicated in the pathophysiology of MDD [4,17,19], though neuropathological evidence of MAP in the brains of subjects with MDD is inconsistent [4,18,148,239]. One neuropathological study found a positive association between suicidality and both MAP density and microglial quinolinic acid expression [17]. In rats, chronic psychological stress promotes MAP in the prefrontal cortex, amygdala, and hippocampus [19]. Recent meta-analysis in MDD patients confirmed elevation of serum levels of proinflammatory cytokines, such as interleukin 6 (IL-6) and TNFα [23,240]. Multiple *in vitro* studies of various neurological conditions showed that MAP and proinflammatory cytokines could increase BBB permeability [4,38-40,168,184,241] (Figures 1 and 2) (Table 1). BBB hyperpermeability may in turn increase crosstalk between innate and adaptive immunity, thereby resulting in further upregulation of MAP and brain cytokine production in a positive feedback loop [242]. MAP can activate iNOS [8,11,26,27], increase ROS synthesis [28], and promote COX2 expression within the neurovascular unit [4]; these factors may increase BBB permeability *in vitro *[38,53]. MAP and proinflammatory cytokines can release and activate matrix metalloproteinases (MMPs) [38,39,168], which have been shown *in vitro* to disrupt BBB endothelial tight junction proteins and increase BBB opening [38,39,168,184]. Serum MMP-9 levels have been shown to correlate with depressive symptom severity in humans (as assessed by the Hamilton Depression Scale) [97]. Highly reproducible *in vitro* data showed that proinflammatory cytokines (TNFα, IL-1β, interferon γ (IFNγ)) can cause a dose-dependent increase in BBB permeability by inducing expression of intercellular adhesion molecule 1 (ICAM-1) on the luminal surface of BBB endothelial cells in animals [243-249] and humans [250,251]. One neuropathological study found a significant increase in the ICAM-1 expression in the deep white matter of the dorsolateral prefrontal cortex in MDD relative to controls [172]. Another study showed SSRIs can reduce vascular endothelial expression and serum levels of both ICAM-1 and vascular cell adhesion molecule 1 (VCAM-1) [173]. Thus, increased BBB endothelial cell expression of adhesion molecules may be one mechanism by which BBB hyperpermeability occurs in MDD [174,252] (Figure 2). However, contrary to this interpretation, a separate postmortem study has shown decreased expression of VCAM-1 and ICAM-1 in the orbitofrontal cortex in depressed subjects compared with non-depressed controls [174]. Increased TNFα production occurring after acute myocardial infarction is associated with an increased risk of MDD and BBB endothelial hyperpermeability [241]. *In vitro* animal studies showed that TNFα could reduce mitochondrial density and impair mitochondrial oxidative metabolism, leading to increased ROS synthesis [11,253]. Several lines of human [65,113-121] and animal [65,122] evidence implicate mitochondrial abnormalities in MDD. *In vitro* data mechanistically link mitochondrial abnormalities to oxidative injury-related vascular abnormalities [219] (Figures 1 and 2). Thus, proinflammatory cytokines may also induce depression and increase BBB permeability by promoting oxidative stress and impairing mitochondrial functions. The relevance of these mechanisms to MDD, however, remains unproven.

Bradykinin is a polypeptide that mediates inflammation, vasodilation, and increased capillary permeability. Human data of bradykinin alterations in MDD are limited to evidence of functional single nucleotide polymorphisms of the bradykinin receptor B2 gene (BDKRB2) [158] (Table 1). LPS-induced depressive-like behavior in mice was associated with upregulation of bradykinin activity and bradykinin B1 receptor expression [159]; further, selective bradykinin B1 receptor antagonists improved depression-like behavior [159]. Activation of bradykinin and its inducible B1 and constitutively expressed B2 receptors induces inflammation, promotes oxidative injury, and increases BBB permeability [160] (Figures 1 and 2). Bradykinin activation can augment the astroglial NFκB pathway-mediated IL-6 production, which may increase BBB permeability [168,184]. Bradykinin activation can also stimulate phospholipase A2 activity, which in turn enhances arachidonic acid release and its metabolism, leading to increased malondialdehyde [12] and NO production [38] that may increase BBB permeability. Activation of B2 receptor increases endothelial Ca^2+^ influx, which can activate pro-oxidant enzymes involved in ROS synthesis [38,168,184]. Increased ROS production can increase BBB permeability and its susceptibility to the harmful effects of bradykinin [12]. *In vitro* human studies showed that inflammation-related upregulation of BBB endothelial bradykinin B1 receptor expression could increase BBB permeability [160].

Glutamatergic hyperfunction may contribute to neurovascular dysfunction in MDD (Figure 2 and Table 1). Numerous experimental paradigms such as, brain proton magnetic resonance imaging, postmortem brain investigations, and CSF studies, have documented glutamatergic hyperfunction in persons with MDD [4,161,162]. Neuroinflammation may contribute to hyperglutamatergia in a positive feedback loop through several potential mechanisms, which include: (a) inhibition and reversal of astroglial excitatory amino acid transporter-mediated glutamate reuptake function (this process mediates more than 90% of glutamate uptake [254]); (b) stimulation of microglial synthesis of quinolinic acid, which can promote synaptosomal glutamate release and increase astroglial glutamate and d-serine release; and (c) upregulation of MAP expression of X_c_ antiporter system, which increases microglial glutamate release [4]. Postmortem investigations of *N*-methyl-d-aspartate receptors (NMDARs) subunit expression in the brains of MDD subjects compared with those of non-depressed controls show (a) an increase or no change of NR1 subunit expression in the hippocampus [107-109], (b) an increase of NR2A and NR2B subunit expression in the hippocampus [107,108], (c) a decrease or no change in NR1 subunit expression in the prefrontal cortex [110,111], (d) a decrease of NR2A and NR2B subunit expression in the prefrontal cortex [110], and (e) an increase of NR2A subunit expression in the lateral amygdalae [255]. Binding of excess glutamate to its dysregulated BBB endothelial ionic NMDARs and metabotropic glutamate receptors (mGluRs) can increase intracellular Ca^2+^ level-dependent oxidative stress and BBB permeability via increasing Ca^2+^ influx and release from endoplasmic reticulum stores, respectively [38,40,159,256]. Animal data showed that NMDAR activation facilitates free radical production such as ONOO^-^[38,40,256] (Figures 1 and 2). Administration of glutamate receptor antagonists has been shown to attenuate NMDAR-induced oxidative stress [40]. Animal studies showed that oxidative stress in turn can alter cerebral endothelial NMDAR subunit composition and upregulate NR1 subunit expression [40], thus setting up a positive feedback loop that increases BBB endothelium vulnerability to both glutamate excitotoxicity and oxidative stress [40]. Alteration of endothelial NMDAR subunit compositions may also reduce cerebral blood flow, as physiologic activation of endothelial NMDAR may activate eNOS and increase endothelial-derived NO [256]. BBB breakdown may also increase CNS glutamate levels via disruption of endothelial-bound glutamate efflux transporters [44]; in turn, hyperglutamatergia may heighten BBB susceptibility to the harmful effects of bradykinin. Administration of glutamate receptor antagonists can block bradykinin-induced endothelial Ca^2+^ rise [38]. Thus, BBB hyperpermeability, increased endothelial NMDAR expression, and increased CNS glutamate levels may contribution to neuronal dysfunction in MDD.

Mast cells are tissue-bound granulated cells most commonly found in the skin and gastrointestinal tract. They, like basophils, contain high levels of histamine and heparin. In the brain, mast cells are particularly abundant in the hypothalamic region. Mast cell activation has been associated with MDD [169] (Table 1). Approximately 40% to 70% of persons with mastocytosis, an uncommon and heterogeneous syndrome characterized by increased mast cell density, exhibit depressive symptoms [257]. Increased corticotropin-releasing hormone (CRH) secretion may contribute to mast cell activation associated with MDD [168,170,171]. Experimental evidence suggests that mast cells can cause inflammation [170], modulate BBB permeability [170], and facilitate NMDAR-induced neuronal excitotoxicity [170] (Figure 2). Mast cell activation can release inflammatory substances (for example, IL-6, TNFα, vascular endothelial growth factor) and stimulate vascular endothelial cell adhesion molecule expression [170]. These molecules can disrupt BBB integrity and enhance inflammatory cell transmigration into the brain [170].

### Future Directions

Human and animal studies are needed to evaluate the validity of the BBB dysfunction hypothesis and to explore the mechanistic links between oxidative stress, eNOS uncoupling, and neuroinflammation and neurovascular unit dysfunction with BBB hyperpermeability in MDD. Future postmortem studies investigating the relationship between neurovascular unit dysfunction with BBB hyperpermeability and MDD should focus primarily on the neuroanatomical regions where astroglial loss and MAP have been documented in MDD brains such as anterior mid/cingulate cortex, prefrontal cortex, amygdala, and white matter [4]. Developing methods with increased sensitivity to detect and quantitate subtle BBB hyperpermeability in MDD are likely to be informative [37]. These methods might utilize fluorescent dyes in animal models of depressive-like behavior similar to those developed for *in vivo* imaging of specific neurovascular elements in animal models of various neurological disorders associated with neurovascular dysfunction [43]: sulforhodamine 101 dye, Ca^2+^ sensitive dyes, glial fibrillary acidic protein (GFAP), AQP4 (astroglia), CX3C chemokine receptor 1 (CX3CR1) (microglia), dextran-conjugated dyes, alpha SMA-RFPcherry (pericytes), dextran dyes, Tie2 (vasculature) and Thy1 (neurons) [43]. A promising neuroimaging modality for visualizing MAP in humans with psychiatric illnesses is PET imaging utilizing microglial peripheral benzodiazepine receptor (also known as translocator protein) C11-PK11195 radioligand [4,258-260]. We suspect that various neurovascular processes particularly those promoting endothelial (and potentially astroglial) eNOS dysfunction may emerge as key targets for cellular and molecular research in MDD. Adequately powered randomized controlled trials investigating the effects of anti-inflammatory agents and antioxidants in MDD [4,90] should also assess their effects on cerebral microvascular endothelial functions (for example, by utilizing techniques that measure peripheral vascular dilatory response [182] and cerebral perfusion [90]), as well as the relationship between the extent of endothelial dysfunction and the severity of depressive symptoms.

## Conclusions

Neurovascular dysfunction with BBB hyperpermeability may occur in MDD. Cumulative clinical and experimental evidence implicates oxidative stress, eNOS uncoupling, and reduced endothelial NO levels in the pathophysiology of peripheral vascular endothelial dysfunction associated with MDD. Our theoretical integration of the human and animal data links oxidative stress, eNOS uncoupling, low endothelial NO levels, and neuroinflammation to putative neurovascular and BBB abnormalities in MDD. If future studies confirm their relevance to the pathophysiology of MDD, novel agents correcting these abnormalities may prove to be effective treatment strategies.

## Abbreviations

AQP4: Aquaporin 4; BH2: Dihydrobiopterin; BH4: Tetrahydrobiopterin; CBF: Cerebral blood flow; COX2: Cyclooxygenase 2; CRH: Corticotropin-releasing hormone; CSF: Cerebrospinal fluid; CT: Computed tomography; EEG: Electroencephalogram; eNOS: Endothelial nitric oxide synthase; EAAT: Excitatory amino acid transporter; Fc: Immunoglobulin constant region; H2O2: Hydrogen peroxide; HO-: Hydroxyl radical; ICAM-1: Intercellular adhesion molecule 1; IL: Interleukin; iNOS: Inducible nitric oxide synthase; MAP: Microglial activation and proliferation; MDD: Major depressive disorder; MRI: Magnetic resonance imaging; mGluR: Metabotropic glutamate receptor; MMPs: Matrix metalloproteinases; NAD(P)H: Nicotinamide adenosine dinucleotide phosphate; Na+/K+ ATPase: Sodium-potassium adenosine triphosphates; NFκB: Nuclear factor κB; NMDAR: *N*-methyl-D-aspartate receptor; NO: Nitric oxide; ONOO-: Peroxynitrite; O2-: Superoxide; PET: Positron emission tomography; PLA2: Phospholipase A2; RNS: Reactive nitrogen species; ROS: Reactive oxygen species; RUR: Relative uptake ratio; SOD-1: Superoxide dismutase 1; SPECT: Single photon emission computed tomography; SSRI: Selective serotonin reuptake inhibitor; Th: T helper; TNFα: Tumor necrosis factor α; TReg: CD4^+^CD25^+^FOXP3^+^ T regulatory; VCAM-1: Vascular cell adhesion molecule 1.

## Competing interests

The authors declare that they have no competing interests.

## Authors’ contributions

SN, DMP conceived and designed the research; SN, DMP wrote the manuscript; SN, DMP, AN, OD, DZ, revised the manuscript for important content; SN, DMP, AN, OD, DZ, performed literature searches and gathered data for the review; all authors read and approved the final version of the manuscript for submission.
